# OP76 Direct Costs Associated With HER2+ Breast Cancer Care In A Colombian Healthcare Institution: A Real-World-Evidence Study

**DOI:** 10.1017/S0266462325101311

**Published:** 2025-12-29

**Authors:** Manuel González, Sandra Arauchan, Sergio Beltran, José Lobatón, Danis Rojas, Javier Ospina, Daniel Samacá

## Abstract

**Introduction:**

Economic restrictions can affect breast cancer healthcare attention; therefore, it is essential to identify the main economic factors that impact the health system’s budget. This study identified and quantified monetarily the events generating direct costs associated with the care of HER2+ breast cancer according to stage of the disease in a Colombian healthcare institution.

**Methods:**

This retrospective observational study included women over 18 years of age with a diagnosis of HER2+ breast cancer identified through International Classification of Diseases, 10th Revision codes present in medical records between January 2018 and December 2022. Resources were organized into the categories of laboratory tests, medical consultations, procedures, diagnostic imaging, supplies, radiotherapy, and medications. The monetary valuation was carried out using the UPC Sufficiency, RIPS, SOAT, and SISMED prices databases for 2023. A micro-costing technique and a third-payer perspective were employed. The average and total care costs by baseline stage were evaluated.

**Results:**

During the study period, 289 patients with HER2+ breast cancer were identified (age 53 years, standard deviation 11.7) with the following disease stages: in situ (n=2 patients), early (n=68), locally advanced (n=165), metastatic (n=41), and not reported (n=13). The total cost for managing the cohort was USD13,012,354, with the cost for the in situ stage being USD11,953 versus USD7,465,033 for locally advanced. Compared with treating patients in the situ stage, the average treatment cost was 4.8 times higher for early cancer, 7.6 times higher for locally advanced cancer, and 13.8 times for metastatic disease. The costs associated with medications represented 83 percent of the total, followed by procedures with eight percent.

**Conclusions:**

The comprehensive care of patients with HER2+ breast cancer reflects a significant economic burden for the healthcare system, showing an increase in average costs in the advanced stages of the disease. These results highlight the importance of early diagnosis from an economic perspective on resources used for patients with HER2+ breast cancer to guide decision-making and achieve efficient resource allocation.

